# Clinical characteristics of anaphylaxis and risk factors for severe reactions in children: a single-center experience

**DOI:** 10.3389/fped.2026.1769228

**Published:** 2026-04-21

**Authors:** Wenyu Wang, Yueyun Shang, Hui Zhang, Shuang Ba, Tongqiang Zhang

**Affiliations:** 1 Department of Critical Care Medicine (Machang Campus), Children's Hospital, Tianjin University/Tianjin Children's Hospital, Tianjin, China; 2 Tianjin Key Laboratory of Birth Defects for Prevention and Treatment, Tianjin, China; 3 Department of Respiratory (Beichen Campus), Children's Hospital, Tianjin University/Tianjin Children's Hospital, Tianjin, China; 4 Department of Respiratory, Shanxi Children's Hospital (Shanxi Women and Children Health Hospital), Taiyuan, Shanxi, China

**Keywords:** anaphylaxis, drug allergy, food allergy, risk factors, severe anaphylaxis

## Abstract

**Background:**

This study aimed to analyze the clinical characteristics, severity, and risk factors for severe outcomes of anaphylactic reactions in pediatric patients.

**Methods:**

This study examined 107 pediatric anaphylaxis cases from September 2020 to July 2025. A retrospective analysis was performed on demographic data, clinical features, laboratory results, and allergen triggers. Patients were categorized by reaction severity for comparative analysis. Multivariate logistic regression was used to identify risk factors for severe anaphylaxis.

**Results:**

Among the 107 children included in this study, male 64 cases (59.81%) and female 43 cases(40.18%), ages ranged from 1 month to 15 years. Food was the most common trigger (62.61%, 67/107). In children under 1 year, egg allergy was most prevalent (37.50%, 6/16), while in those over 6 years, fruit allergy predominated (37.50%, 15/40). Antibiotic allergy was the most common drug-related trigger across all age groups (44.44%, 13/26). Severe anaphylaxis was more prevalent in children over 6 years old (36/47 *vs.* 34/60, *p* = 0.031), males (34/47 *vs.* 30/60, *p* = 0.019), and those with drug allergies (16/47 *vs.* 10/60, *p* = 0.037). Multivariate logistic regression identified male sex, age over 6, drug allergies, and short onset time as significant risk factors for severe anaphylaxis.

**Conclusions:**

Food is the primary trigger for anaphylaxis in children, with allergen types and severity varying by age. Male children, those over 6 years old, children with drug allergies, and those with rapid-onset reactions are at higher risk for severe anaphylaxis and require focused clinical attention.

## Introduction

Anaphylaxis refers to severe, potentially life-threatening systemic allergic reactions, characterized by rapid multi-organ involvement and unpredictable, often fatal outcomes (1, 2). This condition can affect various systems, including the skin, respiratory, circulatory, and gastrointestinal systems, with circulatory failure and complete airway obstruction posing the greatest risk of mortality (3). In recent years, the incidence of anaphylaxis has gradually increased, becoming a global health concern (4). In the United States, the incidence of severe allergies rose from 14.2 to 28.6 cases per 100,000 people annually between 2005 and 2014 (5). In Sweden and Denmark, the incidence among children was reported at 32 and 26.8 cases per 100,000 annually, respectively (6, 7). In Hong Kong, China, the incidence of pediatric anaphylaxis increased from 2.46 to 6.63 cases per 100,000 annually from 2001 to 2015 (8). These rising rates among children highlight the growing concern. However, in China, systematic research on pediatric anaphylaxis remains limited, and clinical practice faces challenges such as insufficient identification, diagnosis, and treatment of the condition. This article presents a retrospective study of 107 pediatric anaphylaxis cases observed at our hospital, analyzing clinical features, allergic triggers, and potential risk factors for severe reactions. The findings aim to provide valuable insights for pediatricians in the management of these cases.

## Methods

### Research subject

**Figure 1:**
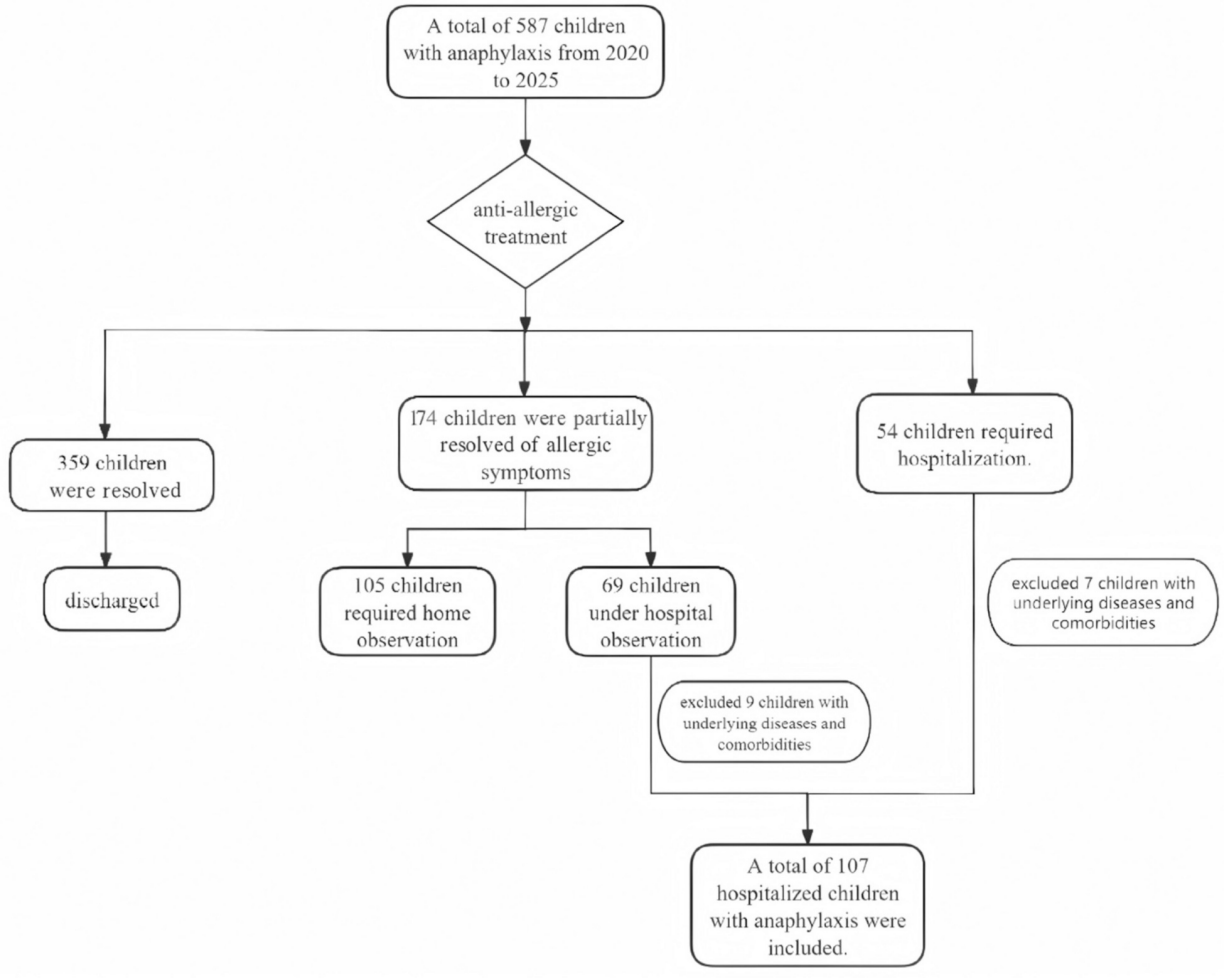
The specific inclusion process for the children.

A clinical data analysis of children diagnosed with anaphylaxis who were hospitalized between September 2020 and July 2025 was performed. The specific inclusion process for the children is as shown in the following Figure 1. Clinical information, including age, gender, history of allergic diseases, suspected allergens, clinical characteristics, laboratory results, treatment protocols, and follow-up outcomes, was examined.The study was conducted in accordance with the Declaration of Helsinki and was approved by the Ethics Committee of Tianjin Children's Hospital(W-2025-028). This study was conducted as a retrospective analysis, with the identities of pediatric participants anonymized to ensure confidentiality. The requirement for informed consent was waived by the institutional ethics review board.

### Diagnostic criteria and severity classification

In accordance with the 2024 WAO guidelines for diagnosing anaphylaxis (9), a child is considered to have a high likelihood of an allergic reaction if they meet any one of the following three criteria: 1) No Known Allergen Exposure:Sudden onset of an ilness (minutes to several hours) with skin/mucosal and either: Respiratory involvement, Cardiovascular involvemert. 2) Likely or Known Allergen Exposure, sudden onset of two or more of following: a) skin or mucosal tissues:urticaria, flushing, erythema, facial swelling; Infants may also have mottling, lip, tongue, or oropharyngeal swelling, severe throat tightness, difficulty swallowing; Infants may also have repetitive lip licking. b) Respiratory:wheezing, increased work of breathing, hypoxemia, cough, dyspnea; Laryngeal:stridor, voice change; Infants may also have a hoarse cry; c) Cardiovascular:hypotension, syncope, dizziness, unexplained change in mental status; Infants may also have persistent unexplained tachycardia; d) Gastrointestinal:severe crampy abdominal pain, repetitive vomiting, diarrhea. 3) Acute hypotension, bronchospasm, or laryngeal involvement occurring within minutes to hours following exposure to known or suspected allergens, even in the absence of typical skin involvement.

Anaphylaxis is classified into three degrees—mild, moderate, and severe—according to these guidelines (10, 11). This classification corresponds to the grading system in the latest expert consensus on severe anaphylaxis (12). Mild allergic reactions (Grade 1) are characterized by skin or mucosal symptoms along with mild involvement of other systems. Moderate reactions (Grades 2 and 3) involve moderate symptoms extending beyond the cutaneous system, affecting respiratory, cardiovascular, gastrointestinal, and neurological systems, with the patient remaining conscious and maintaining normal systolic blood pressure. Severe anaphylaxis (Grades 4 and 5) includes signs such as cyanosis, oxygen saturation <92%, apnea, hypotension (≤70 mmHg for infants under 1 year; ≤70 mmHg + 2 × age for children aged 1 to 10 years; ≤90 mmHg for those aged 11 to 17 years), circulatory failure, arrhythmias, bradycardia, cardiac arrest, altered consciousness, or even loss of consciousness.

### Statistical analysis

Data processing and analysis were performed using SPSS version 27.0 statistical software. Normally distributed continuous variables are expressed as mean ± standard deviation (mean ± SD), while non-normally distributed variables are presented as medians(IQR, 25th−75th percentile).For comparisons between groups with normally distributed data, the t-test was used; for comparisons where the data did not follow a normal distribution or had unequal variances, the Mann–Whitney U-test is employed. Categorical data are presented as rates or percentages (%), and group comparisons were analyzed using the *χ*^2^ test or Fisher's exact test. Variables with a *p* < 0.1 in univariate analysis, along with clinically important factors, were entered into a multivariate logistic regression model. Multivariate logistic analysis was utilized to identify risk factors for severe anaphylaxis. A *p* < 0.05 was considered statistically significant.

## Results

### General condition of patients with anaphylaxis

This study included 107 hospitalized children diagnosed with anaphylaxis, consisting of 64 males (59.81%) and 43 females (40.18%). Ages ranged from 1 month to 15 years, with the majority being over six years old (70 cases, 65.42%). The time elapsed between exposure and the onset of symptoms (EAOS time) (<30 min) was observed in 57 patients (53.27%). The severity of allergic reactions was classified as mild/moderate in 60 cases (56.07%) and severe in 47 cases (43.93%). The average values for laboratory tests were: white blood cell (WBC) count 15.65 ± 6.94 × 10^9^/L, eosinophils 0.7 (0.2,1.27) × 10^9^/L, immunoglobulin E (IgE) 293.30 (138.80,430.00) IU/mL, and interleukin-6 (IL-6) 24.00 (7.78,37.30) pg/mL (Table 1).

**Table 1:** General condition of anaphylaxis.

Characteristic	Cases	Percentage (%)
Gender		
Male	64	59.81
Female	43	40.18
Age		
<1 year old	17	15.89
1–6 years old	20	18.69
>6 years old	70	65.42
Family history of allergic	15	14.02
Allergy history	77	
Food allergy	7	7.60
Drug allergy	5	5.43
Allergic rhinitis/asthma	29	31.52
Eczema/Dermatitis	51	55.43
EAOS time (min)*		
<30 min	57	53.27
>30 min	50	46.73
Severity		
Mild/Moderate	60	56.07
Severe	47	43.93
Laboratory information		
WBC (10^9/L)	15.65 ± 6.94	
Eosinophil (10^9/L)	0.7 (0.2, 1.27)	
IgE (IU/mL)	293.30 (138.80, 430.00)	
IL-6 (pg/mL)	24.00 (7.78, 37.30)	

EAOS (The time elapsed between exposure and the onset of symptoms) time:the 30-minute cutoff was predefined based on the well-recognized “golden 30 min” principle in anaphylaxis management. The data are expressed as mean ± SD or median (IQR,25th-75th percentile).

### Analysis of triggers and age distribution in patients with anaphylaxis

**Figure 2:**
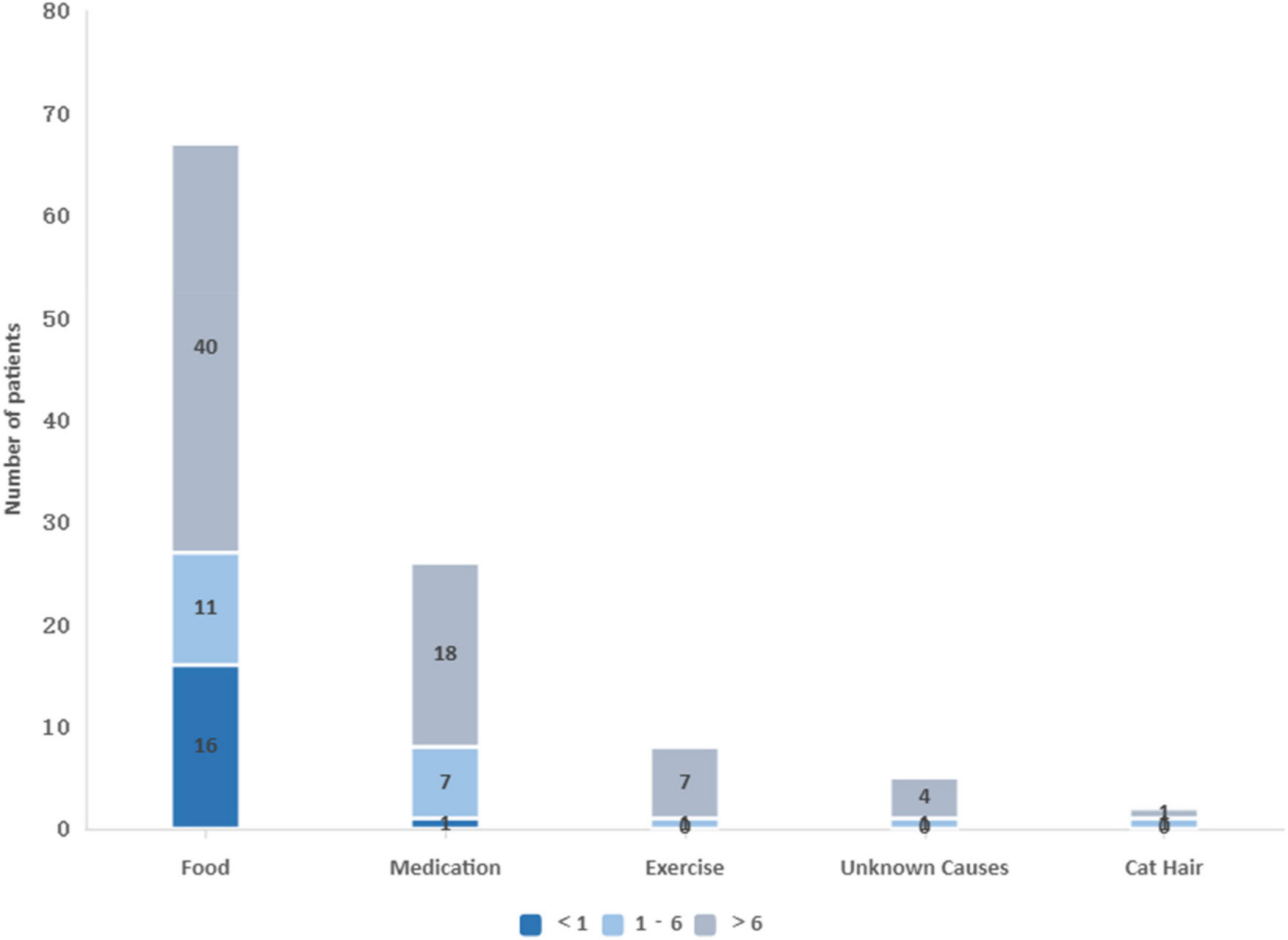
Allergic triggers in different age groups.

Food allergies were the most common cause of anaphylaxis, accounting for 67 cases (62.61%), followed by drug allergies (26 cases, 24.30%), cat hair allergy (1 case, 0.93%), exercise-induced allergies (8 cases, 7.47%), and unexplained allergies (5 cases, 4.67%) (Figure 2).

Among food allergies, the top three allergens were fruits (18 cases, 26.86%), wheat products (11 cases, 16.41%), and nuts (9 cases, 13.43%). In children under one year, egg allergies were most common (6 cases, 37.50%), followed by milk/formula allergies (4 cases, 25.00%). For children over six years old, fruit allergies (15 cases, 37.50%) and nut allergies (7 cases, 17.50%) were most frequent. Among drug allergies in children over six years, antibiotics were the most commonly reported (8 cases, 44.44%). Both exercise-induced and unexplained allergies were also more common in this age group, at rates of 10.14% (7/69) and 5.80% (4/69), respectively (Table 2).

**Table 2:** Analysis of triggers and age distribution in patients with anaphylaxis.

Trigger	Age range	Cases	<1	1–6	>6
Food	4 months −15 years	67	16	11	40
Eggs	6 months - 8 months	6	6	0	0
Milk/Formulas	3 months - 9 years	6	4	1	1
Seafood	1 year −13 years	4	0	2	2
Noodles	7 months −12 years	11	3	4	4
Rice	8 years 6 months	1	0	1	1
Fruits	11 months−13 years	18	1	2	15
Nuts	1 years−15 years	9	0	2	7
Vegetables	6 months−11 years	8	2	0	6
Medication	11 months−16 years	26	1	7	18
Antibiotics	11 months−15 years	13	1	5	8
Antivirals	8 years 4 months	2	0	1	1
Antipyretic and detoxifying drugs	5 years−13 years	7	0	2	5
Sedatives	7 years 8 months	1	0	0	1
Pepsin powder	12 years 2 months	1	0	0	1
Traditional Chinese Medicine	6 years−16 years	2	0	0	2
Cat hair	4 years 7 months	1	0	1	0
Exercise	5 years−14 years	8	0	1	7
Unknown causes	5 years−12 years	5	0	1	4

### Comparison of clinical characteristics according to the severity

**Figure 3:**
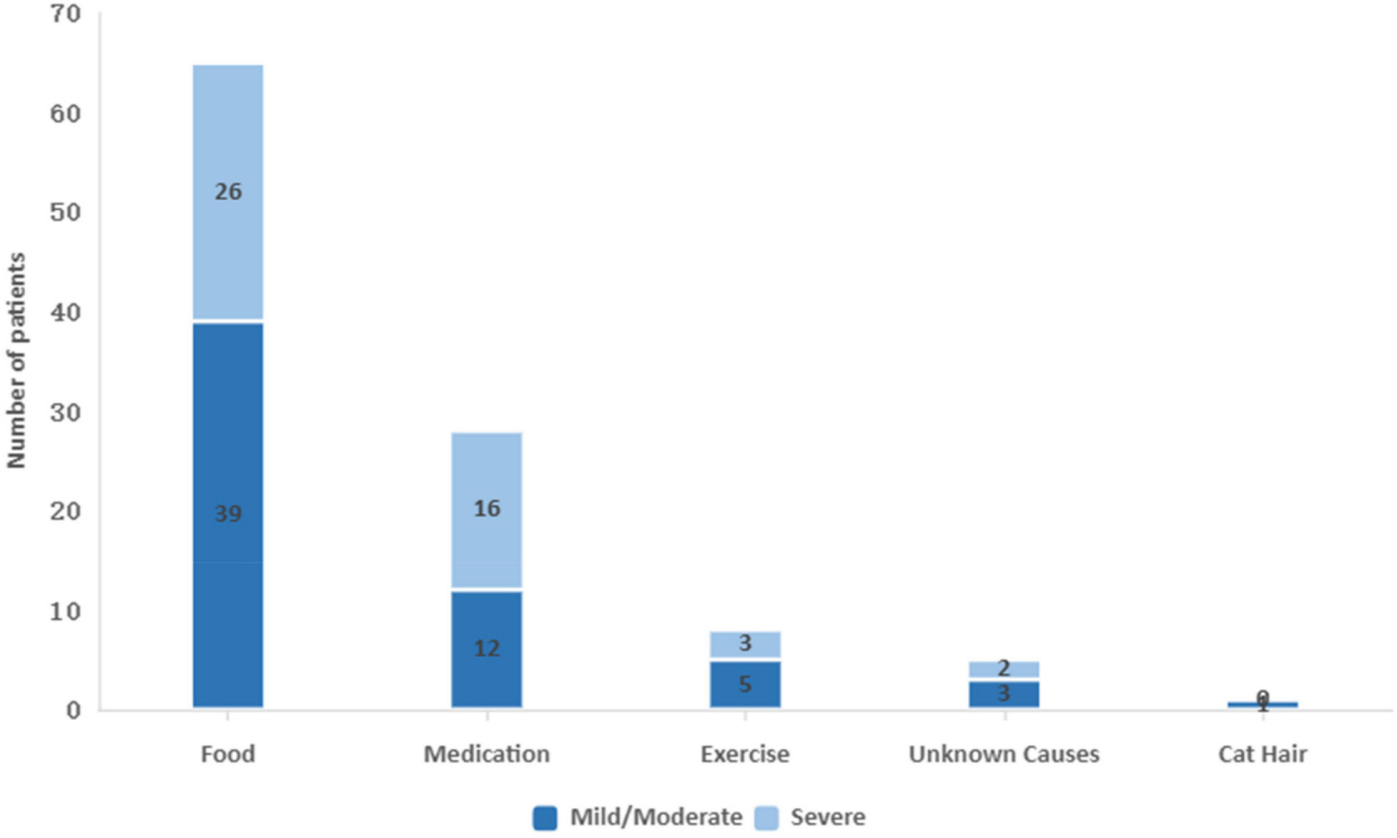
Composition of triggers for anaphylaxis among different groups.

In this study, 47 of the 107 anaphylaxis cases were classified as severe. Among these, 16 were drug-induced, 26 were food-induced, 3 were exercise-induced, and 2 had unknown causes (Figure 3).

The male proportion in the severe anaphylaxis group was 34 (72.34%), significantly higher than the mild/moderate group, which had 30 males (50.00%) (*p* < 0.05). Of those with severe anaphylaxis, 36 cases (76.60%) were over six years old, compared to 34 cases (56.67%) in the mild/moderate group (*p* < 0.05). The severe anaphylaxis group also had a higher proportion of patients with drug allergies (34.04%, 16/47) than mild/moderate group (16.67%, 10/60) (*p* < 0.05). The proportion of patients with EAOS time <30 min was significantly higher in the severe group than in the mild/moderate group (85.10% vs 28.33%, *p* < 0.05). Myocardial damage occurred in 14 cases (29.78%) of severe anaphylaxis, which were higher than the 7 cases (11.67%) observed in the mild/moderate group (*p* < 0.05). No significant differences were found between the two groups regarding previous allergy history or underlying disease. Additionally, there were no notable differences in the levels of WBC, eosinophils, IgE, or IL-6 between the two groups (Table 3).

**Table 3:** Severity of anaphylaxis.

Characteristic	mild/moderate	Severe	*P*
Gender			0.019
Male	30 (50.00)	34 (72.34)	
Female	30 (50.00)	13 (27.66)	
Age			0.031
<6 years	26 (43.33)	11 (23.40)	
>6 years	34 (56.67)	36 (76.60)	
Allergy history	39 (65.00)	38 (80.85)	0.060
History of diseases	3 (5.00)	5 (10.64)	0.725
Triggers			0.037
Medication	10 (16.67)	16 (34.04)	
Non-medication	50 (83.33)	31 (65.96)	
EAOS time (min)			0.001
<30 min	17 (28.33)	40 (85.10)	
>30 min	43 (71.67)	7 (14.90)	
Previous allergy history*	2 (3.33)	5 (10.64)	0.445
WBC (10^9/L)	15.03 ± 6.07	17.19 ± 6.94	0.067
Eosinophil (10^9/L)	0.30 (0.10, 0.88)	0.50 (0.10, 1.07)	0.537
IgE (IU/mL)	143.18 (54.11, 339.65)	226.28 (48.42, 468.70)	0.374
IL-6 (pg/mL)	24.00 (10.56, 26.75)	27.57 (5.05, 71.00)	0.221
Myocardial injury	7 (11.67)	14 (29.79)	0.019
Elevated creatinine	1 (1.67)	0 (0)	-
Abnormal liver function	0 (0)	1 (2.13)	-

Previous allergy history: There has been a history of Anaphylaxis in the past. The data are expressed as mean ± SD or median (IQR,25th-75th percentile).

### Risk factors for anaphylaxis

The multivaraite analysis included 47 cases. The final model demonstrated good calibration (Hosmer-Lemeshow *χ*^2^ = 3.086, *p* *=* 0.877). The multivariable logistic regression model demonstrated excellent discrimination, with an AUC of 0.882 (95% CI: 0.794–0.970, *p* < 0.001), indicating its ability to distinguish between patients with mild/moderate and severe anaphylaxis. Multivariate logistic analysis revealed that male gender (OR 4.607, 95% CI 1.557–7.913), age over 6 years (OR 3.510, 95%CI 1.014–5.760), and drug allergies (OR 3.696, 95% CI 1.141–6.380) are significant risk factors for severe anaphylaxis. Additionally, a shorter EAOS time to the allergen was identified as an independent risk factor for severe anaphylaxis (OR 5.917, 95% CI 2.825–9.174). Allergy history (OR 2.073, 95% CI 0.854–4.082) and WBC (OR 1.086, 95% CI 0.920–1.508) were not an independent risk factor for severe anaphylaxis. (Table 4).

**Table 4:** Analysis of risk factors for severe anaphylaxis.

Variable	*P*	OR (95%CI)
Gender	0.008	4.607 (1.557–7.913)
Age	0.047	3.510 (1.014–5.760)
Medication	0.043	3.696 (1.141–6.380)
<30 min	0.001	5.917 (2.825–9.174)
Allergy history	0.104	2.073 (0.854–4.082)
WBC	0.078	1.086 (0.920–1.508)

### Treatment and follow-up

Among the 107 cases, 97 were treated with IM epinephrine in the emergency department, including 43 cases (91.49%) in the severe anaphylaxis group and 54 cases (90.00%) in the mild/moderate group. For 16 cases with no response to the first IM epinephrine administration, epinephrine was re-administered intramuscularly at 5 to 15 min intervals (34.00%, 16/47), and the number of repeated injections did not exceed 3 times in our hospital. No biphasic reaction occurred in these cases. Furthermore, 35 cases (74.47%, 35/47) of severe anaphylaxis patients received physiological saline for volume expansion. No fatalities occurred among the severe anaphylaxis cases; however, one patient required invasive mechanical ventilation. After aggressive treatment, the patient was successfully weaned off ventilation and discharged in stable condition.

A follow-up telephone survey was conducted for 47 children with severe anaphylaxis after discharge, with 44 cases followed up within 3 to 6 months (Table 5).

**Table 5:** Follow-up 3 to 6 months post-discharge.

Cases	Detailed information
1	Mild/moderate anaphylaxis reoccurred following discharge.
14	Developed an allergic rash that was alleviated through intermittent use of antihistamines and topical medications.
2	Allergic to “pasta and eggs” showed improvement as they aged; their families reported over the phone that there were no further allergic reactions upon re-exposure to these foods.
27	No significant allergic reactions were observed following discharge.
3	Loss to follow-up

## Discussion

This study conducted a comprehensive analysis of the general conditions, clinical features, and allergic triggers in 107 children diagnosed with anaphylaxis. Four significant risk factors for severe anaphylaxis were identified: gender, age, medications, and EAOS time. These factors are relatively straightforward for children to communicate during medical consultations, which can aid pediatric clinicians in promptly identifying severe cases of anaphylaxis.

Food was identified as the most common trigger of anaphylaxis in children across all age groups, accounting for 62.61%. This finding aligns with reports from both European and Chinese literature, which indicate food as the leading cause of anaphylaxis in children, with prevalence rates of 55.5% and 66%, respectively (13, 14). Given the limited ability of infants to articulate symptoms, diagnosing anaphylaxis in this age group poses a challenge and requires careful attention from pediatric clinicians. In infants, eggs (37.50%, 6/16) and milk/formula (25.00%, 4/16) were the predominant triggers. Other studies also report eggs (53.33%, 8/15) and milk (26.67%, 4/15) as common allergens among infants (80%, 12/15) (14). In Asia, 90% of food-induced anaphylaxis in infants in Japan is triggered by eggs (62.1%), cow's milk (20.1%), and wheat (7.1%) (15). In the United States, peanut and milk allergies are particularly prevalent among infants (16). These differences are believed to be influenced by various factors, including ethnicity, dietary habits, climate, and environmental conditions. Research suggests that while milk and egg allergies typically resolve with age, nut allergies tend to persist (17, 18). Follow-up surveys with parents of infants revealed that as children aged, allergic reactions to eggs and milk/formula diminished, consistent with prior studies. Milk allergy in infants often resolves spontaneously between 12 and 24 months (18). Among children over six years old, severe food allergies remain prevalent, with fruits accounting for 37.50% and nuts for 17.50%. Nut allergies, in particular, are known to be strong triggers for anaphylaxis and are among the primary causes of such reactions in North America and the United Kingdom (19, 20).

Research in Europe has shown that the triggers of anaphylaxis in children shift from food to medication around the age of 10, after which allergic conditions tend to stabilize (21). The proportion of children experiencing anaphylaxis due to medications increases with age (22). The present study found that medication-induced anaphylaxis was more common in children over six years old (69.20%). A large-scale analysis in South Korea of emergency anaphylaxis cases revealed that medications are the most common cause of anaphylaxis in individuals aged seven and older (4). Research has indicated that drug-induced anaphylaxis typically exhibits more severe symptoms, highlighting the need for heightened vigilance among pediatric clinicians (23). In the present study, antibiotics were identified as the most common drug-related anaphylaxis trigger (55.00%). Xin et al. reported that antibiotics account for 53% of drug allergies in children (24). Pediatricians should inquire about previous drug allergies when prescribing antibiotics, particularly for older children.

A retrospective study of 991 pediatric anaphylaxis cases in South Korea found that male children were more frequently affected (66%) than females (34%) (22). Similarly, Linus B et al. analyzed data on severe allergic reactions from 10 European countries, also revealing a higher incidence in male children (65%) (13). Our study showed that the proportion of male children affected (59.80%) was greater than that of female children (40.18%), consistent with the findings of these previous studies. Research suggests that the development of childhood atopic diseases in boys is influenced by various factors, including sex hormone levels and the immune regulatory system (25). A multicenter study in South Korea identified drug allergies and increasing age as significant risk factors for severe anaphylaxis (23). A double-blind food challenge in the Netherlands also indicated that advancing age is a critical predictor of severe anaphylaxis in children (26). An analysis of fatal anaphylaxis cases in the United States found a higher incidence among children over the age of 12 (27). Similarly, in the present study, severe anaphylaxis was more prevalent in children over six years old and those with drug allergies, compared to those with mild or moderate reactions. These findings align with both retrospective and prospective studies conducted abroad.

This study highlights the rapid progression of anaphylaxis, with EAOS time <30min (85.10%, 40/47) in severe group. A double-blind food challenge trial conducted in the Netherlands involving 734 children found that a shorter interval between allergen ingestion and the onset of symptoms was associated with more severe allergic reactions (26). Notably, 14 cases (29.79%) of myocardial damage were observed in the severe anaphylaxis group, compared to 7 cases (11.67%) in the mild/moderate group. This difference may be linked to factors such as systemic vasodilation, fluid extravasation, coronary artery spasm, and inflammatory mediators damaging myocardial cells (28). When children present with myocardial injury or even cardiac arrest, pediatricians should recognize Kounis syndrome early by monitoring ECG abnormalities. The syndrome is related to mast cell activation secondary to allergic reactions (29). In the present study, no significant correlations were observed between previous allergy history, underlying diseases, or laboratory markers such as WBC count, eosinophil percentage, IgE levels, and IL-6, and the severity of anaphylaxis. Similarly, a statistical analysis from South Korea involving 199 adults and children with anaphylaxis found no significant association between these parameters and reaction severity (23). Turner PJ et al. also demonstrated that IgE levels are not related to the severity of anaphylaxis and have no predictive value (30).

Emergency treatment for anaphylaxis involves intramuscular epinephrine, antihistamines, corticosteroids, and saline solution (31). The administration of intramuscular epinephrine significantly reduces mortality in pediatric anaphylaxis cases (32). In the present study, the epinephrine usage rate for hospitalized anaphylaxis cases was 90.65% (97/107), similar to the 91.8% reported in a study of children with anaphylaxis (14). No fatalities occurred in our cohort; which may be attributable to prompt, standardized epinephrine therapy as first-line management for childhood anaphylaxis during admission (9). Following discharge, guardians were instructed to give epinephrine immediately in the event of recurrent severe anaphylaxis. one child required mechanical ventilation but was successfully extubated shortly after hospitalization, resulting in a favorable prognosis and stable follow-up. A 3 to 6 month follow-up for children with severe anaphylaxis revealed that some developed subsequent symptoms such as rashes and other allergic manifestations. However, only one case of mild/moderate anaphylaxis was reported. This may be due to improved discharge education, with explicit guidance on allergen avoidance and pre-hospital emergency management, together with counseling on the risk of biphasic reactions. These measures have helped lower recurrence rates and achieve better clinical outcomes. Communication with parents confirmed that strict food avoidance measures were adopted post-discharge, particularly among parents of older children.

This study has several limitations. First, it primarily involves hospitalized children, suggesting the need for a larger sample size and community-based studies. Second, regional limitations warrant nationwide multicenter research to assess the overall incidence of anaphylaxis. Third, the events-per-variable ratio in our final predictive model was borderline, which may introduce a potential risk of overfitting. Therefore, the findings should further validation in larger, independent cohorts is warranted to confirm the generalizability of our model. Finally, medical history collection may have included inaccuracies or omissions, and provocation tests could not be performed due to the high risk associated with anaphylaxis.

In conclusion, this study confirms that food is the most common trigger for anaphylaxis in children, with variations in allergens and severity across age groups. Pediatricians should particularly focus on male children, older children, those with drug allergies, and those experiencing rapid-onset anaphylaxis to ensure timely intervention.

## Data Availability

The original contributions presented in the study are included in the article/Supplementary Material, further inquiries can be directed to the corresponding authors.
